# Designing and characterizing first Iranian study evaluating serum levels of lithium in patients for population pharmacokinetics (FIRELOLIPOP): baseline and first report

**DOI:** 10.1038/s41598-025-99698-y

**Published:** 2025-05-03

**Authors:** Mohammad Mahdi Khakshoor, Naser Pariz, Vahid Reza Askari, Mohammad Reza Fayyazi Bordbar, Sara Honari, Matin Shirazinia, Vafa Baradaran Rahimi, Mohammad Bagher Naghibi Sistani

**Affiliations:** 1 Department of Electrical Engineering, Faculty of Engineering, Ferdowsi University of Mashhad, Mashhad, Iran; 2 Clinical Research Development Unit, Imam Reza Hospital, Faculty of Medicine, Mashhad University of Medical Sciences, Mashhad, Iran; 3 Psychiatry and Behavioral Sciences Research Center, Mashhad University of Medical Sciences, Mashhad, Iran; 4 Faculty of Medicine, Mashhad University of Medical Sciences, Mashhad, Iran; 5 Department of Cardiovascular Diseases, Faculty of Medicine, Mashhad University of Medical Sciences, Mashhad, Iran

**Keywords:** Bipolar disorder, Lithium, Pharmacokinetics, Therapeutic drug monitoring, Personalized medicine, Psychiatric disorders, Molecular biology, Neuroscience, Psychology, Risk factors, Biologics, Biomarkers, Drug safety, Target validation

## Abstract

Bipolar disorder (BD) is a chronic psychiatric illness characterized by recurrent manic and depressive episodes, leading to significant impairment. Lithium remains a key treatment for BD, particularly in relapse prevention. However, its narrow therapeutic range and inter-individual pharmacokinetic variability necessitate careful dosing. This study aims to establish a suitable platform to investigate the pharmacokinetics of lithium in hospitalized patients with BD in Mashhad, Iran, to optimize therapeutic use and minimize toxicity. This cross-sectional study was conducted at Ibn Sina Hospital, Mashhad, between 2016 and 2022. Hospitalized patients diagnosed with BD and receiving lithium therapy were included. Clinical, demographic, and laboratory data were collected, including lithium serum levels, renal function parameters, and co-administered medications. Advanced data pre-processing techniques were applied to ensure accuracy and facilitate future pharmacokinetic modeling. A total of 701 patients (53.1% male, mean age: 38.0 SD: ± 12.2 years) with 795 hospitalization episodes were analyzed. The mean lithium serum concentration was 0.65 ± 0.30 mEq/L. Thyroid disorders (5.9%) and diabetes (5.6%) were the most common comorbidities. The mean duration of hospitalization during lithium treatment was 21.7 ± 10.8 days. Sodium valproate was the most frequently co-prescribed medication (*n* = 553), followed by lorazepam (*n* = 468) and risperidone (*n* = 458). Lithium dosing showed considerable variability, emphasizing the need for individualized therapeutic strategies. This study provides valuable insights into lithium pharmacokinetics in Iranian BD patients. The findings highlight the necessity of personalized dosing approaches to enhance efficacy and reduce adverse effects. Future research should incorporate pharmacokinetic modeling and machine learning to refine lithium therapy.

**Supplementary Information:**

The online version contains supplementary material available at 10.1038/s41598-025-99698-y.

## Introduction

Bipolar disorder (BD), characterized by alternating episodes of mania and depression, is one of the most severe psychiatric conditions, often leading to significant occupational, cognitive, social, and emotional impairments^1,2^. Clinically, BD is categorized into two main subtypes: type I, marked by distinct manic episodes, and type II, defined by hypomanic episodes alongside major depressive episodes^3^. The global prevalence of BD has increased significantly, rising from 24.8 million cases in 1990 to 39.5 million cases in 2019. Among mental disorders, BD ranks as the fourth leading cause of years lived with disability (YLDs) and disability-adjusted life years (DALYs) across all age groups, following depressive disorders, anxiety disorders, and schizophrenia. Moreover, it ranks as the third cause of DALYs and YLDs rates in the 15–24 years age group^4^.

A manic episode is considered a medical emergency that requires immediate treatment to prevent self-harm or harm to others. The recommended approach for adults experiencing acute manic symptoms involves pharmacological intervention. Several agents, including sodium valproate, lithium, and antipsychotics, have demonstrated sufficient efficacy in managing such episodes^5^. Once the acute mood disturbances subside, most patients require prolonged medical treatment, often lasting several years. Lithium becomes a cornerstone therapy in this stage, known as maintenance treatment. Lithium has shown greater efficacy than sodium valproate in preventing relapses of acute episodes^3,6^. As a result, lithium is recognized as one of the foundational treatments in bipolar disorder management guidelines^7^.

Following lithium oral administration, the immediate-release form is absorbed rapidly and effectively through the upper gastrointestinal tract within 1–6 h^8^. Lithium is mainly absorbed on the jejunum and ileum^9^ and its absorption can be influenced by dietary factors^10^. Once absorbed, lithium dissociates completely and circulates as a free cation in plasma, without binding to plasma proteins^11,12^. Due to its physicochemical properties and small molecular size, lithium distributes extensively across both extracellular and intracellular fluid compartments. However, its movement from the extracellular to intracellular space is relatively slow, attributed to a low diffusion coefficient across cell membranes. The volume of distribution for lithium has been reported to range between 0.8 and 1.2 L/kg^13,14^.

Lithium undergoes minimal metabolic transformation, as it bypasses hepatic metabolism. Approximately 2% of lithium is excreted via bile within 24 h of oral administration^15,16^. The drug is primarily eliminated as a free cation in the urine through glomerular filtration, with approximately 80% of the filtered lithium reabsorbed in the proximal tubule^17–19^. In individuals with normal renal function, including patients with bipolar disorder, the terminal elimination half-life (t½) ranges from 16 to 30 h^20–24^. Lithium clearance demonstrates considerable interindividual variability, with reported rates ranging from 0.6 to 2.4 L/h^14,17^. Several physiological and pathological factors, such as age, total body weight, and renal function, affect total body clearance^25^.

Due to lithium’s narrow therapeutic window, careful monitoring of serum levels is essential to ensure both efficacy and safety. Therapeutic concentrations range from 0.6 to 1.5 mEq/L for acute mania and 0.6 to 0.8 mEq/L for maintenance therapy^26^. Serum levels exceeding 1.5 mEq/L can lead to toxicity, with early symptoms including gastrointestinal disturbances (e.g., nausea, vomiting, diarrhea), followed by neurological complications such as tremor, confusion, slurred speech, and agitation^27–31^. Other lithium side effects include seizures, loss of appetite, muscle weakness, and an increase in urine volume^32^. Therefore, accurate dose adjustment is crucial to minimize adverse effects while maintaining therapeutic benefit^33^.

Safety, efficacy, and accurate dose determination of the drugs are some concerns that have been always exist since drug discovery and development. Population pharmacokinetics (PK) is one of the proposed solutions to overcome these concerns^34^. Population PK means investigating and evaluating the differences in plasma concentrations of the drugs between different people receiving the same standard drug regimen. Age, weight, and gender can be mentioned among the factors examined in these studies. It is important to put these factors together and predict an accurate dose for each person especially for those drugs that have a narrow therapeutic window. In this way, it is possible to reduce the costs of therapeutic drug monitoring, drug side effects due to dose increase, or response reduction due to dose reduction^35^.

Several physiological and pathological factors influence lithium pharmacokinetics, including age, renal function, body weight, circadian rhythm, genetic factors, cardiovascular conditions, pregnancy, and lactation^36,37^. Studies have also indicated that genetic factors are linked to bipolar disorder, particularly with regard to the age of onset. These findings suggest that genetic factors may influence the progression of the disease and play a significant role in shaping its course, leading to important differences in modeling^37^. Most of these factors affect the lithium PK through their impact on kidney function^38^. Despite the existing body of evidence regarding the PK of lithium, there are some controversies surrounding this issue^39^. For instance, the optimal lithium dose for managing BD type II is unclear^40^. Levels effective for acute mania are not always suitable for type II BD, and many clinicians believe that lower serum levels, typically 0.4–0.8 mmol/L, may still be adequate. Additionally, while the lithium therapeutic range has been extensively studied, there is debate over the best dosing regimen. Some experts recommend a once-daily dosage, while others favor dividing the daily dose into two or three smaller doses. Those supporting a once-daily regimen highlight lithium’s mean half-life of 24 h^8,28,41–43^. Ultimately, lithium dosing should prioritize clinical response over achieving specific serum levels, as lower levels do not necessarily indicate subtherapeutic effects^39^.

Given these complexities, we established the first Iranian registry dedicated to studying lithium pharmacokinetics in hospitalized patients with bipolar mood disorder. This article presents the baseline demographic and clinical characteristics of enrolled patients and outlines the methods and design of the FIRELOLIPOP study (First Iranian Registry Evaluating Lithium Levels for Population Pharmacokinetics), marking an initial step toward individualized lithium therapy in Iranian clinical practice.

## Methods

### Method

This is a cross-sectional study conducted between 2016 and 2022 at Ibn Sina Hospital in Mashhad, Iran, on hospitalized patients with bipolar disorder. Mashhad, the second most populous city in Iran, has a population of over 3 million and is located in the east of the country. Ibn-Sina Hospital is also a reference center in the field of psychiatry and a referral center for bipolar patients in the east of the country. All procedures have been approved by the Mashhad University of Medical Sciences Ethical Committee (Ethical permission code: IR.MUMS.MEDICAL.REC.1401.485, permission date: 2022–11–08, Approval ID: 4011114). All methods were performed in accordance with the Mashhad University of Medical Sciences Ethical Committee guidelines and regulations (Ethical permission code: IR.MUMS.MEDICAL.REC.1401.485, permission date: 2022–11–08, Approval ID: 4011114).

### Inclusion criteria

**Fig. 1:**
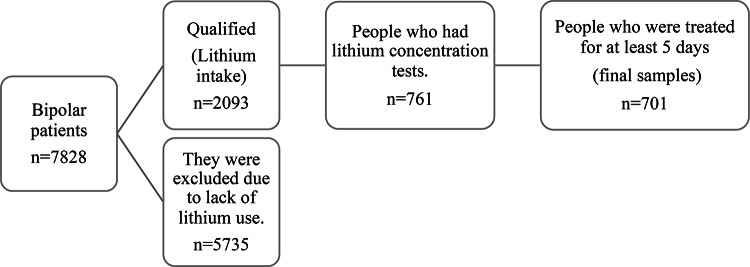
Patients’ inclusion and exclusion criteria.

Patients with a confirmed diagnosis of BD who were prescribed lithium were included in the study. Patients were excluded if their serum lithium levels had not been measured or if they were hospitalized for less than five days. Patients hospitalized for fewer than 5 days were excluded to ensure that serum lithium concentrations reflect steady-state levels, which are typically reached after ~ 5 days of regular dosing given lithium’s half-life of approximately 24 h. The diagnosis of bipolar disorder in all patients was based on the ICD-10 code, which was made by a specialist physician. According to the International Classification of Diseases, 10th Revision (ICD-10), BD is coded under F31.0 to F31.9, encompassing a spectrum of clinical presentations including hypomanic, manic (with or without psychotic features), depressive, mixed episodes, and remission states^44^. All details are given in Fig. 1.

### Data collection

First, a list of patients was compiled based on ICD-10 codes between March 2016 and March 2022. These codes include nine different disease categories, ranging from F31.1 to F31.9, as defined by the ICD-10 classification. Subsequently, blood and urine test data—comprising approximately 100 tests—were extracted. However, since some tests had been performed on only a small number of patients and were not considered meaningful, only 28 relevant tests with a sufficient number of records were included in the dataset. These 28 tests consist of lithium, WBC, RBC, Hct, MCH, MCV, MCHC, PMN, Hb, lymphocytes, monocytes, platelets, eosinophils, potassium, sodium, creatinine, urea, SGPT (ALT), SGOT (AST), ALP, calcium, TSH, RDW-CV, Fe, TIBC, folic acid, serum magnesium, and albumin.

All medications used by patients were extracted from the hospital registry system to check for their concurrence with lithium use. After initial reviews, the most important and most frequently used of them, 61 items, were entered into the data table. These medications, along with their standard dosages, are listed in Table 1.

**Table 1:** Drugs and calculated standard doses.

Drug	Dose	Drug	Dose	Drug	Dose	Drug	Dose
Alprazolam	0.5 mg	Clonidine	0.2 mg	Levetiracetam	500 mg	Propranolol	10 mg
Amantadine	100 mg	Clozapine	25 mg	Levothyroxine	0.05 mg	Quetiapine	25 mg
Amitriptyline	10 mg	Cobalamin	1000 mcg/ml	Lithium	300 mg	Risperidone	1 mg
Aripiprazole	5 mg	Cyproterone	50 mg	Lorazepam	1 mg	Sertraline	50 mg
Atorvastatin	20 mg	Divalproex	250 mg	Magnesium hydroxide	250 mg	Thiamine	100 mg
Azithromycin	250 mg	Fluconazole	150 mg	Metformin	500 mg	Tizanidine	4 mg
Biperiden	2 mg	Fluoxetine	10 mg	Methadone	5 mg	Topiramate	25 mg
Bismuth	120 mg	Flupentixol	20 mg	Midazolam	5 mg/ml	Trihexyphenidyl	2 mg
Bupropion	75 mg	Fluphenazine	25 mg	Naproxen	250 mg	Valproate	200 mg
Calcium-D	500 mg-200 IU	Folate	0.4 mg	Olanzapine	5 mg	Vitamin C	1000 mg
Carbamazepine	200 mg	Furosemide	40 mg	Omeprazole	20 mg	Vitamin D	50,000 IU
Cefalexin	500 mg	Gabapentin	100 mg	Oxybutynin	5 mg	Vitamin E	100 IU
Chlordiazepoxide	5 mg	Glibenclamide	5 mg	Pantoprazole	20 mg	Zolpidem	5 mg
Chlorpromazine	25 mg	Haloperidol	5 mg	Perphenazine	2 mg		
Citalopram	20 mg	Haloperidol	0.5 mg	Phenobarbital	60 mg		
Clonazepam	1 mg	Lamotrigine	25 mg	Promethazine	50 mg		

Then, the patient’s clinical and demographic data were collected using a predetermined checklist, including height, weight, age, gender, marital status, personal and family history of mental illness, drug, alcohol, and smoking history, blood pressure, medical and surgical history, first episode of mental illness, number of days of hospitalization, address of birth and residence, nationality, and other such items. Variables such as BMI, GFR, and several other composite variables were also added to the data.

### Extracting, integrating, and controlling data quality

The medical staff carefully collected all data, and various stages of data engineering were performed under the supervision of an artificial intelligence expert. This process included various parts, including quality review, merging different data tables, effective and understandable data arrangement, process simplification, completion of incomplete data, and removal of existing anomalies. All the characteristics and different periods of tests and medications were recorded in one Excel row and for a specific time period for each patient so that in the future, other specialists who do not know data science or database management could easily use this data to expand their knowledge and research.

Also, pre-processing was done to understand the data better and help remove existing obstacles and shortcomings. Advanced tools and software such as Python^®^ and RapidMiner^®^ were used for pre-processing, joining, monitoring, and ensuring the quality of the data to minimize any bias and implausible data. Python were extensively utilized for various tasks, including aligning different graphs, consolidating multiple tables into a single unified table, verifying and validating data placement, detecting noise and resolving it with expert consultation, as well as performing data structuring and engineering. Additionally, RapidMiner software facilitated a deeper understanding of the dataset through diverse visualization techniques, which proved valuable for writing articles and advancing the research. Furthermore, the software greatly assisted researchers in refining the dataset by detecting subtle errors and noise, performing data transformations, identifying outliers, and enhancing data quality through its advanced preprocessing and machine learning algorithms.

### Statistical analysis

Continuous data were summarized using mean, minimum, maximum, and standard deviation, while categorical data were presented as frequencies and percentages. Chi-squared and Fisher’s exact tests were employed to compare demographic characteristics across different types of BD based on ICD-10 classification. A p-value of less than 0.05 was considered statistically significant.

## Results

**Fig. 2:**
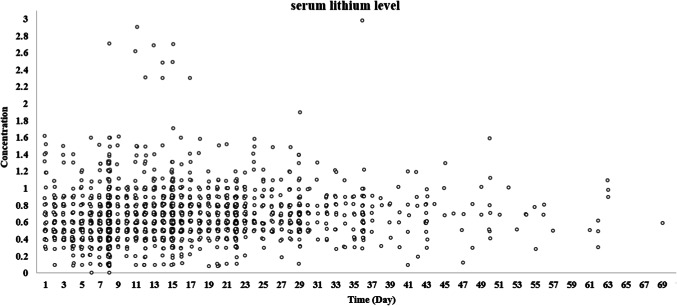
Concentration-time graph of serum lithium level for all admissions (*n* = 795).

Between March 2016 and March 2022, a total of 7828 patients (12196 episodes) were admitted to our center with a diagnosis of BD, of whom only 2093 patients were treated with lithium during their hospitalizations. Lithium concentration results were only recorded in the data repository after March 2019, which limited the study sample to this period. Ultimately, 701 distinct patients with 795 admission episodes were included in this study (Supplementary Tables S1-S4). The concentration-time graph of serum lithium level for all admissions is shown in Fig. 2.

### Patients’ characteristics

Of the 701 patients included, 372 (53.1%) were male, with a mean age of 38.02 years (± 12.21) (Table 2). A total of 361 patients (51.5%) were married, 225 (32.1%) were single, and 422 (60.2%) were residents of Mashhad. Regarding diagnostic categories, 154 patients (22.0%) were classified under F31.1, 153 (21.8%) under F31.2, 42 (6.0%) under F31.3 to F31.8, and 352 (50.2%) under F31.9. The mean body mass index (BMI) of the patients was 25.91 kg/m^2^ (± 5.71), with a mean height of 166.03 cm (± 9.60) and a mean weight of 71.39 kg (± 16.41). The estimated glomerular filtration rate (eGFR), calculated using the Cockcroft-Gault, CKD-EPI, and MDRD formulas, was 100.60 mL/min/1.73 m^2^ (± 28.67), 93.47 mL/min/1.73 m² (± 17.83), and 80.80 mL/min/1.73 m^2^ (± 18.55), respectively.

**Table 2:** Patient characteristics - quantitative values.

Title		Age	Day	Real_Day	Height	Weight	BMI	GFR		
								MDRD	CKD	Cockcroft-Gault
Total	**Min**	10	6	6	140	35	14.5	39.23	43.63	42.13
	**Max**	78	73	73	195	141	56.5	264.29	156.25	217.50
	**Avg**	38.02	27.71	21.66	166.03	71.39	25.91	80.80	93.47	100.60
	**Sd**	12.21	11.40	10.80	9.60	16.41	5.71	18.55	17.83	28.67
Male	**Min**	13	6	6	150	42	15.4	39.23	43.63	42.13
	**Max**	78	67	66	195	135	44.1	264.29	156.25	217.50
	**Avg**	38.98	28.54	21.55	172.71	74.84	25.05	86.12	98.26	106.99
	**Sd**	12.43	10.99	10.37	7.05	14.57	4.40	19.42	17.23	29.96
Female	**Min**	10	6	6	140	35	14.5	42.53	48.56	45.77
	**Max**	70	73	73	185	141	56.5	140.41	135.04	202.58
	**Avg**	36.93	26.78	21.80	159.39	67.96	26.76	74.84	88.10	94.36
	**Sd**	11.87	11.77	11.27	6.79	17.39	6.66	15.48	16.94	25.87
F31.1	**Min**	15	8	6	141	40	14.5	45.52	51.18	52.97
	**Max**	75	62	56	195	141	56.5	152.42	139.55	200.00
	**Avg**	38.13	26.10	21.12	165.06	72.67	26.73	79.39	92.11	101.66
	**Sd**	12.61	10.56	9.94	9.96	17.94	6.47	17.08	17.63	28.07
F31.2	**Min**	16	6	6	150	42	16.5	42.55	50.00	50.76
	**Max**	73	65	65	190	121	48.5	140.41	129.29	184.72
	**Avg**	39.03	30.49	23.60	167.50	72.03	25.62	80.63	93.53	101.95
	**Sd**	11.76	10.60	10.80	9.70	15.74	5.12	16.40	17.16	26.93
F31.9	**Min**	10	7	7	140	35	15.2	39.23	43.63	42.13
	**Max**	67	73	73	185	135	51.1	264.29	156.25	217.50
	**Avg**	37.11	27.53	21.07	165.48	70.78	25.86	81.68	94.27	100.74
	**Sd**	11.66	11.58	10.89	9.29	16.28	5.69	20.08	18.05	29.47
F31.3-8	**Min**	15	6	6	149	37	14.8	44.29	51.45	51.52
	**Max**	78	67	66	185	93	33.2	130.65	134.04	163.27
	**Avg**	41.62	25.07	21.62	168.87	69.67	24.43	79.20	91.47	89.96
	**Sd**	15.45	13.44	12.20	9.48	13.57	4.48	17.17	18.61	27.86

(Real_Day = Days on lithium treatment), (ICD10 : F31.1 = Bipolar affective disorder, current episode manic without psychotic symptoms, F31.2 = Bipolar affective disorder, current episode manic with psychotic symptoms, F31.3 = Bipolar affective disorder, current episode mild or moderate depression, F31.4 = Bipolar affective disorder, current episode severe depression without psychotic symptoms, F31.5 = Bipolar affective disorder, current episode severe depression with psychotic symptoms, F31.6 = Bipolar affective disorder, current episode mixed, F31.7 = Bipolar affective disorder, currently in remission, F31.8 = Other bipolar affective disorders, F31.9 = Bipolar affective disorder, unspecified)

The most frequent comorbidities identified were thyroid abnormalities (41 patients, 5.9%), diabetes mellitus (39 patients, 5.6%), hypertension (27 patients, 3.9%), cardiovascular diseases (12 patients, 1.7%), dyslipidemia (11 patients, 1.6%), neurological disorders (8 patients, 1.1%), liver disease (5 patients, 0.7%), cancer (2 patients, 0.3%), gynecological disorders (2 patients, 0.3%), and kidney disease (1 patient, 0.1%) (Table 3).

**Table 3:** Patient characteristics - nominal and present or absent values.

Title	Values	Tot	M	F	F31.1	F31.2	F31.9	F31.3-8
First episode	Mania	252	144	108	64	53	127	8
	Dep	99	44	55	22	19	52	6
	None	350	183	165	67	80	173	28
Marriage	M	361	203	158	83	80	178	20
	S	225	121	104	51	44	115	15
	W	17	3	14	4	6	5	2
	D	76	34	42	12	20	40	4
	None	22	11	11	4	3	14	1
Addiction	Me	82	60	22	18	23	32	9
	Am	15	8	7	3	7	5	0
	P	38	28	10	6	13	17	2
	N	566	276	290	127	110	298	31
H_Addiction		135	96	39	27	43	54	11
H_Smoking		252	175	77	58	56	119	19
H_Alcohol consumption		50	28	22	10	15	21	4
H_Suicide		92	43	49	13	16	53	10
Resident city		422	221	201	101	87	208	26
Resident province		609	326	283	136	130	307	36
Iranian nationality		690	368	322	152	152	345	41
PPH_OCD		35	23	12	6	8	19	2
PPH_ADHD		23	13	10	3	4	14	2
PPH_Dep		19	10	9	1	4	11	3
PPH_PD		9	8	1	0	1	7	1
PPH_MR		4	2	2	2	1	1	0
PPH_PTSD		6	5	1	2	0	4	0
H_Trauma & head injury		99	61	38	11	20	59	9
H_Seizure		81	44	37	16	11	47	7
H_Thyroid disorder		41	14	27	13	8	17	3
H_Diabetes		39	19	20	12	6	18	3
H_Blood pressure		27	15	12	6	7	13	1
H_Liver disease		5	1	4	1	1	3	0
H_stroke		3	2	1	1	0	0	2
H_Kidney disease		1	1	0	0	0	1	0
H_Heart attack		4	3	1	0	0	4	0
H_Heart disease		9	5	4	1	1	7	0
H_CVD		12	7	5	1	1	10	0
H_Surgery		28	17	11	3	6	18	1
H_Internal disease		24	6	18	10	8	5	1
H_HLP		11	8	3	6	2	3	0
H_Neurological disease		8	5	3	2	3	3	0
H_Women’s disease		2	0	2	0	1	1	0
H_Urological disease		1	1	0	0	1	0	0
H_Otolaryngology		0	0	0	0	0	0	0
H_Cancer		2	1	1	0	0	2	0
H_Eye disease		0	0	0	0	0	0	0
F.H_bipolar disorder		63	27	36	17	12	29	5
F.H_Mental disorder 1		160	88	72	30	36	80	14
F.H_Mental disorder 2		78	40	38	21	15	41	1
F.H_Depression		35	16	19	7	7	16	5
F.H_Epilepsy		9	3	6	2	2	4	1

(H = History) – (F.H = Family History) – (PPH = Past psychiatric history) – (First episode; Dep = Depression, Mix = Mix episode) – (Marriage; M = Married, S = Single, D = Divorce, W = Widow) – (Addiction; Me = Methadone, Am = Amphetamine, P = Positive but type unknown, N = Negative) – (CVD = Cardiovascular disease) – (HLP = Hyperlipidemia).

### Patients’ psychiatric characteristics

The initial presentation of the disease was mania in 253 patients (71.8%) of the 351 patients evaluated for this criterion, while 100 patients (28.2%) initially presented with depression. Substance addiction was documented in 135 patients (19.3%), including methadone addiction in 82 patients (60.7%), amphetamine addiction in 15 patients (11.1%), and other unspecified addictions in the remaining cases. Smoking was reported by 252 patients (36.0%), and 50 patients (7.1%) consumed alcohol. Regarding past psychiatric history (PPH), obsessive-compulsive disorder (OCD) was the most common concurrent condition, affecting 35 patients (5.0%), followed by attention-deficit hyperactivity disorder (ADHD) in 23 patients (3.3%) and post-traumatic stress disorder (PTSD) in 6 patients (0.9%). Head trauma was reported in 99 patients (14.1%), and 81 patients (11.6%) experienced at least one seizure episode. Among first-degree family members, 63 patients (9.0%) had a relative with BPD, 35 patients (5.0%) had a relative with depression, and nine patients (1.3%) had a relative with a history of epilepsy. Of all the patients included, 92 patients (13.1%) had a history of suicide attempts, and four patients (0.6%) were noted to have an intellectual disability.

Different types of BD, as classified by ICD-10, showed significant differences in terms of sex (*P* = 0.032) and history of head trauma (*P* = 0.017). Other factors did not significantly differ across groups, including history of OCD (*P* = 0.926), ADHD (*P* = 0.592), intellectual disability (*P* = 0.487), PTSD (*P* = 0.695), seizure (*P* = 0.155), thyroid abnormalities (*P* = 0.427), diabetes mellitus (*P* = 0.462), hypertension (*P* = 0.922), cardiovascular disease (*P* = 0.246), dyslipidemia (*P* = 0.109), smoking (*P* = 0.475), and alcohol use (*P* = 0.419).

### Patients’ characteristics during hospitalization

The mean length of hospital stay was 27.7 days (± 11.4), whereas the mean duration of hospitalization during lithium treatment was 21.7 days (± 10.8). A total of 1,850 lithium serum concentrations were recorded, which the technique for serum lithium level monitoring was atomic absorption, with a mean serum lithium concentration of 0.65 mEq/L (± 0.30) (Table 4). The most commonly requested laboratory evaluations included complete blood count (CBC) (1,386 tests), serum potassium (960 tests; mean = 4.03 mEq/L, SD = 0.40), serum sodium (921 tests; mean = 137.79 mEq/L, SD = 3.07), serum creatinine (888 tests; mean = 0.96 mg/dL, SD = 0.17), alanine transaminase (ALT) (888 tests; mean = 25.49 U/L, SD = 25.68), serum urea (887 tests; mean = 28.32 mg/dL, SD = 11.87), aspartate transaminase (AST) (875 tests; mean = 23.35 U/L, SD = 23.74), alkaline phosphatase (ALP) (784 tests; mean = 183.07 U/L, SD = 82.15), serum calcium (752 tests; mean = 9.34 mEq/L, SD = 0.49), and serum thyroid-stimulating hormone (TSH) (173 tests; mean = 3.98 µIU/mL, SD = 6.38).

**Table 4:** Patients’ laboratory tests.

Tests	Count	Period	Min	Max	Avg	SD
Lithium	1850	12	0	3	0.65	0.30
WBC (White Blood Cells)	1386	20	2.1	19.7	7.73	2.59
RBC (Red Blood Cells)	1386	20	3	6.7	4.54	0.56
Hct (Hematocrit)	1386	20	24.4	53.6	39.78	4.26
MCH (Mean Corpuscular Hemoglobin)	1386	20	15.1	43.5	29.43	2.86
MCV (Mean Corposcular Volume)	1386	20	55.8	122.3	88.19	6.94
MCHC (Mean Corpuscular Hemoglobin Concentration)	1386	20	23.9	39.3	33.34	1.59
PMN (Polymorphonuclear neutrophils)	1386	20	33	89	64.14	9.93
Hb (Hemoglobin)	1385	20	5.9	19.6	13.28	1.72
Lymph (lymphocytes)	1385	20	6	63	27.98	9.32
Mono (Mononucleosis)	1383	20	1	18	4.25	1.71
PLT (Platelet)	1375	20	72	467	218.23	68.30
EOS (Eosinophil)	1374	20	1	11	3.65	1.55
K (Potasium)	960	26	3	5.5	4.03	0.40
Na (Sodium)	921	27	122	149	137.79	3.07
Creatinine	888	10	0.4	2	0.96	0.17
ALT or SGPT (Alanine aminotransferase)	888	11	2	231	25.49	25.68
Urea	887	10	7	97	28.32	11.87
AST or SGOT (aspartate aminotransferase)	875	11	7	402	25.35	23.74
ALP (Alkaline phosphatase)	784	10	52	1199	183.07	82.15
Ca (Calcium)	752	4	7.7	10.8	9.34	0.49
TSH (Thyroid stimulating hormone)	173	3	0.1	50.9	3.98	6.38
RDW-CV (Red blood cell distribution width)	113	18	11.8	19	13.90	1.48
Fe (Iron)	49	2	18	156	79.14	32.71
TIBC (Total iron binding capacity)	46	2	257	708	374.28	80.50
Folic Acid	45	2	2.1	120.8	15.72	18.58
MGS (Serum Magnesium)	23	1	1.9	2.6	2.17	0.19
Albumin	12	1	3.5	4.8	4.20	0.43

It is important to note that, based on the doctor’s prescription, each test may be repeated multiple times throughout the patient’s treatment period. For the serum lithium concentration test, the highest number of repetitions recorded for an individual was 12 instances. For other tests, the most frequent repetitions were observed for serum sodium (27 instances), serum potassium (26 instances), and CBC tests (20 instances). Detailed information about the frequency of each test is provided in Table 4. For all tests, the minimum number of prescriptions was one.

The average lithium dose prescribed was 41.05 × 300 mg (± 30.14) (Table 5). The most prevalent drug concurrently prescribed with lithium was sodium valproate (*n* = 553; mean = 68.69 × 200 mg, SD = 61.34), followed by lorazepam (*n* = 468; mean = 28.15 × 1 mg, SD = 24.09), risperidone (*n* = 458; mean = 65.75 × 1 mg, SD = 57.13), haloperidol (*n* = 366; mean = 4.30 × 5 mg, SD = 5.47), quetiapine (*n* = 297; mean = 97.28 × 25 mg, SD = 114.09), biperiden (*n* = 294; mean = 19.35 × 2 mg, SD = 20.05), midazolam (*n* = 294; mean = 3.00 × 5 mg/ml, SD = 3.14), clonazepam (*n* = 241; mean = 22.28 × 1 mg, SD = 23.91), olanzapine (*n* = 207; mean = 34.49 × 5 mg, SD = 35.07), gabapentin (*n* = 205; mean = 49.34 × 100 mg, SD = 62.84), naproxen (*n* = 200; mean = 28.29 × 250 mg, SD = 33.23), promethazine (*n* = 196; mean = 3.41 × 50 mg/ml, SD = 3.95), propranolol (*n* = 141; mean = 36.54 × 10 mg, SD = 35.31), clozapine (*n* = 109; mean = 54.94 × 25 mg, SD = 70.94), and aripiprazole (*n* = 106; mean = 41.80 × 5 mg, SD = 45.61).

**Table 5:** Medications taken by patients.

Drugs	Persons							Dose(*n*)		
	Tot	M	F	F31.1	F31.2	F31.9	F31.3-8	Quantity	Avg	SD
Lithium 300 (mg)	701	372	329	154	153	352	42	28,775	41.05	30.14
Valproate 200.0 (mg)	553	304	249	124	121	280	28	37987.2	68.69	61.34
Lorazepam 1.0 (mg)	468	238	230	102	104	240	22	13,174	28.15	24.09
Risperidone 1.0 (mg)	458	257	201	95	102	240	21	30112.6	65.75	57.13
Haloperidol 5.0 (mg)	366	207	159	74	83	190	19	1572	4.30	5.47
Divalproex 250.0 (mg)	315	145	170	69	67	165	14	8433	26.77	34.14
Quetiapine 25.0 (mg)	297	162	135	58	61	156	22	28,893	97.28	114.09
Biperiden 2.0 (mg)	294	174	120	58	63	160	13	5690	19.35	20.05
Midazolam 5.0 (mg)	294	163	131	68	67	142	17	883	3.00	3.14
Clonazepam 1.0 (mg)	241	154	87	41	51	125	24	5369.8	22.28	23.91
Olanzapine 5.0 (mg)	207	110	97	45	49	101	12	7140.1	34.49	35.07
Gabapentin 100.0 (mg)	205	146	59	34	53	103	15	10,115	49.34	62.84
Naproxen 250.0 (mg)	200	125	75	40	54	92	14	5658	28.29	33.23
Promethazine 50.0 (mg)	196	108	88	47	40	104	5	668.6	3.41	3.95
Propranolol 10.0 (mg)	141	71	70	36	26	66	13	5152	36.54	35.31
Clozapine 25.0 (mg)	109	81	28	22	26	54	7	5988	54.94	70.94
Aripiprazole 5.0 (mg)	106	39	67	26	24	47	9	4431	41.80	45.61
Clonidine 0.2 (mg)	86	64	22	16	26	38	6	507.1	5.90	5.80
Folate 0.4 (mg)	86	28	58	19	26	38	3	5058.5	58.82	77.57
Bismuth 120.0 (mg)	74	49	25	12	19	39	4	506	6.84	8.74
Metformin 500.0 (mg)	72	34	38	21	19	30	2	2157	29.96	32.83
Atorvastatin 20.0 (mg)	70	36	34	24	14	29	3	1255	17.93	12.64
Methadone 5 (mg)	60	48	12	9	16	29	6	3826	63.77	76.95
Fluphenazine 25.0 (mg)	57	39	18	10	8	39	0	75	1.32	0.60
Cyproterone 50 (mg)	51	24	27	10	11	28	2	748	14.67	13.69
Sertraline 50.0 (mg)	49	27	22	7	11	25	6	743	15.16	20.73
Carbamazepine 200.0 (mg)	45	18	27	9	11	22	3	1354	30.09	35.02
Omeprazole 20.0 (mg)	45	20	25	14	11	16	4	610	13.56	11.30
Haloperidol 0.5 (mg)	44	28	16	10	14	17	3	549	12.48	15.19
Lamotrigine 25.0 (mg)	43	27	16	8	13	17	5	2118	49.26	81.02
Zolpidem 5.0 (mg)	43	38	5	6	7	30	0	225	5.23	4.38
Fluconazole 150.0 (mg)	42	2	40	10	11	21	0	122	2.90	1.76
Flupentixol 20.0 (mg)	42	27	15	6	3	32	1	54	1.29	0.45
Pantoprazole 20.0 (mg)	41	24	17	8	11	20	2	654	15.95	16.43
Azithromycin 250.0 (mg)	37	26	11	11	5	18	3	211	5.70	6.40
Cefalexin 500.0 (mg)	35	25	10	6	8	20	1	505	14.43	9.55
Levothyroxine 0.05 (mg)	34	11	23	12	4	16	2	1062	31.24	26.01
Vitamin C 1000.0 (mg)	34	16	18	6	11	16	1	113.6	3.34	4.68
Citalopram 20.0 (mg)	32	21	11	4	2	25	1	150	4.69	3.95
Magnesium hydroxide 250 (mg)	32	11	21	9	6	14	3	43.6	1.36	1.00
Chlorpromazine 25.0 (mg)	28	14	14	6	4	18	0	359	12.82	15.81
Calcium-D 500(mg)-200(IU)	21	8	13	8	5	8	0	360	17.14	15.60
Topiramate 25.0 (mg)	20	9	11	5	6	8	1	1525	76.25	90.79
Fluoxetine 10.0 (mg)	19	13	6	4	4	9	2	345	18.16	33.09
Levetiracetam 500.0 (mg)	18	9	9	7	6	4	1	261	14.50	17.92
Amitriptyline 10.0 (mg)	17	8	9	5	0	12	0	307	18.06	22.34
Trihexyphenidyl 2.0 (mg)	16	9	7	3	2	10	1	241.5	15.09	14.22
Cobalamin 1000 (µg)	15	10	5	4	4	6	1	60	4.00	3.93
Perphenazine 2.0 (mg)	13	5	8	5	3	4	1	152	11.69	9.95
Tizanidine 4.0 (mg)	13	11	2	2	3	5	3	97	7.46	5.96
Bupropion 75.0 (mg)	12	5	7	2	0	6	4	86.3	7.19	4.59
Chlordiazepoxide 5.0 (mg)	12	11	1	2	1	9	0	80	6.67	7.33
Alprazolam 0.5 (mg)	10	6	4	2	0	7	1	42	4.20	4.38
Oxybutynin 5.0 (mg)	10	3	7	4	2	4	0	75	7.50	7.80
Glibenclamide 5.0 (mg)	9	4	5	3	4	2	0	44	4.89	4.51
Thiamine 100.0 (mg)	8	6	2	2	0	6	0	215	26.88	30.88
Furosemide 40.0 (mg)	7	3	4	3	0	4	0	84.5125	12.07	10.90
Vitamin D50000 (IU)	7	2	5	0	3	4	0	28	4.00	2.98
Vitamin E100.0 (IU)	6	1	5	0	2	3	1	48	8.00	8.39
Amantadine 100.0 (mg)	5	1	4	3	0	2	0	19	3.80	5.11
Phenobarbital 60.0 (mg)	3	1	2	0	1	2	0	151.6	50.53	44.41

## Discussion

The current study represents the first of its kind conducted in Iran, specifically in Mashhad, named *First Iranian Study Evaluating Serum Levels of Lithium in Patients for Population Pharmacokinetics (FIRELOLIPOP)*. Several studies have previously been conducted in Iran, including national-level investigations^45–50^ and local studies based specifically in Mashhad^51,52^. However, these studies primarily focused on other topics, such as the simultaneous analysis of risk factors and the evaluation of treatment efficacy through randomized controlled trials. The unique aspects of our study include the large sample size, the extensive range and variety of extracted features, and the meticulous approach to data filtering and organization.

Globally, lithium is recognized as one of the most effective and widely used treatments for managing BD^53–55^. Its efficacy in preventing relapse has been well established^56^. However, as highlighted in prior research^19,43,57^, the use of lithium is complicated by its narrow therapeutic window, the necessity of long-term use for many patients, and the need for regular monitoring of various parameters. These challenges contribute to reduced adherence and declining prescription rates. For instance, it has been reported that less than 35% of BD patients worldwide are currently treated with lithium^58^. Similarly, our study found that only about 25% of bipolar patients admitted to Ibn Sina Hospital in Mashhad were treated with lithium.

Despite these challenges, lithium remains a first-line treatment for BD^59^. A key objective of this study was to establish a comprehensive database to support data science and artificial intelligence-based modeling for analyzing lithium’s treatment process and predicting its concentration-time stability.

In line with the findings of Malhi et al.^43^, which suggest that optimal maintenance concentrations lie in the lower end of the therapeutic range (0.6–0.8 mmol/L), our study found an average serum lithium concentration of 0.65 mmol/L (SD ± 0.3), reinforcing current treatment guidelines.

Diagnostic coding further supported lithium’s primary use in managing manic symptoms: 307 patients (44%) carried ICD-10 codes F31.1 and F31.2, indicating manic without or with psychotic symptoms. These results are consistent with prior research confirming lithium’s potential efficacy for acute mania^55,60,61^. On the other hand, lithium appears less effective in depressive episodes, as noted by other previous studies^56,62^, and reflected in our data—only 24 patients (under 4%) had ICD-10 codes corresponding to depressive states (F31.3–F31.5).

Another key benefit of lithium is its anti-suicidal effect^55^, attributed in part to its neuroprotective mechanisms^63^. Studies show lithium substantially reduces suicide risk^43,62^. In our study, 92 patients (13%) had a history of suicide attempts, and 47 patients (7%) experienced suicidal thoughts. This data underscores the importance of lithium’s protective effects for this group, representing 20% of the study population. Furthermore, the remaining 80% of patients without a history of suicidal attempts or thoughts may have benefited from the positive effects of lithium.

Another unique aspect of this study is the data collected on the concomitant use of more than 60 drugs during lithium treatment. Examining the interactions between these drugs and lithium will be a key goal for future research. For example, past studies have evaluated the combined use of lithium and valproate, finding this combination to be generally beneficial or neutral^51,63,64^. In our dataset, over 80% of patients received either valproate or its derivative Divalproex^®^ in combination with lithium, making this drug family the most commonly co-prescribed with lithium. Similarly, antipsychotics such as olanzapine (*n* = 207) and phenothiazines like perphenazine and chlorpromazine (*n* = 40) were commonly prescribed, consistent with findings from Iranian studies^46^. Our data also capture vitamin supplementation patterns noted previously^49^, allowing for future subgroup analyses.

Renal function monitoring is critical in long-term lithium therapy^63,65^. To address this, we calculated GFR using MDRD, CKD-EPI, and Cockcroft–Gault formulas, providing a multi-faceted view of kidney function that will inform future pharmacokinetic models.

Genetic factors play an essential role in bipolar disorder, as shown in several studies^46,53,59^. Although genetic sequencing data is not available in our study, the recorded family history of bipolar disorder and depression provides a valuable proxy for exploring genetic and familial influences. Notably, 283 patients (40%) had a first- or second-degree relative with bipolar disorder, depression, or another psychiatric condition—highlighting the relevance of familial factors in this population. Neuroimaging and biomarker studies^55,60^ have provided valuable insight into lithium’s effects on brain function, though such data were not available here. Nonetheless, emerging tools such as machine learning^61^ may offer less invasive alternatives, facilitating early diagnosis and treatment optimization while improving patient comfort and reducing costs.

Lithium’s narrow therapeutic window remains its main limitation. Pharmacokinetic-based modeling offers a promising solution to address this challenge. The goal of pharmacokinetics is to study the time course of drug and metabolite concentrations, use relevant data to predict experimental results, and ultimately build appropriate models to interpret such data. In pharmacokinetics, data are analyzed using a mathematical representation of a part or whole organism^66–68^. In fact, PK models show the movement and fate of a drug in a biological system after administration^68^. Therefore, one of the most important methods used to guide lithium therapy is the population pharmacokinetic approach, which takes into account inter- and intra-individual variability in predicting lithium doses. Many studies have conducted in the field of PK^68,69^. Previous studies^69,70^ were also benefited from pharmacokinetic modeling with less data than the data in this study. Thus, one of our main objectives was to create a database capable of supporting advanced lithium PK modeling and predictive analytics in future studies.

### Limitations

Adhering to ethical and legal considerations was a priority for this study. However, the process of obtaining approvals from the University of Medical Sciences, acquiring permits, and extracting patient data took approximately two years, delaying progress. Additional limitations included missing data, technological challenges in recording and collecting information, and inconsistencies in data recording across different periods. The lack of systematic blood and urine test recording before 2019 significantly reduced the volume of available data, limiting our analysis to post-2019 records.

One of the limitations of this study was the inability to consider genetic factors due to the lack of facilities for obtaining such tests. However, an innovative approach for future studies would be to combine geographical, environmental, and familial factors with genetic data to explore their interrelationships. Although genetic data could not be obtained in this study, examining the aforementioned factors and referencing studies that have explored the relationship between these factors and genetics could provide valuable insights.

### Objectives

The study aimed to extract, arrange, and pre-process a wide range of features to facilitate pharmacokinetic modeling using artificial intelligence in future studies. Given the challenges in extracting specific data, especially in Iran and other parts of the world, this study seeks to provide a robust dataset that can shorten and simplify modeling processes using machine learning techniques. Ultimately, this effort aims to reduce treatment duration, minimize costs, and enhance lithium concentration stability in patients with bipolar disorder.

In collaboration with the Faculty of Engineering and the University of Medical Sciences, this study aimed to integrate engineering and medical perspectives. This interdisciplinary approach is expected to advance scientific and technological progress in understanding and treating bipolar disorders and other mental illnesses, which remain significant global health burdens.

### Future work

Future studies will focus on leveraging artificial intelligence, data mining, supervised and unsupervised learning methods, and tools like RapidMiner and Python. These approaches will be combined with key PK parameters such as area under the curve (AUC) and area under the moment curve (AUMC) to develop advanced models for pattern recognition and lithium pharmacokinetics (*First Iranian Study Evaluating Serum Levels of Lithium in Patients for Population Pharmacokinetics (FIRELOLIPOP)*).

## Electronic supplementary material

Below is the link to the electronic supplementary material.

Supplementary Material 1.

## Data Availability

The datasets used and/or analyzed during the current study are available from the corresponding author (V.R.A.) upon reasonable request.
