# Supplementary material for: Designing and characterizing first Iranian study evaluating serum levels of lithium in patients for population pharmacokinetics (FIRELOLIPOP): baseline and first report
Source: Sci Rep. 2025 May 3;15:15514. doi: 10.1038/s41598-025-99698-y (PMC12049548; doi:10.1038/s41598-025-99698-y)
Supplement: Supplementary file 1 — Supplementary Material 1. [file 41598_2025_99698_MOESM1_ESM.docx]

| Title | | Age | Day | Real_Day | Height | Weight | BMI | GFR | | |
| --- | --- | --- | --- | --- | --- | --- | --- | --- | --- | --- |
|  |  |  |  |  |  |  |  | **MDRD** | **CKD** | **Cockcroft-Gault** |
| Tot | **Min** | 10 | 6 | 6 | 140 | 35 | 14.2 | 39.23 | 43.63 | 42.13 |
|  | **Max** | 78 | 73 | 73 | 195 | 141 | 56.5 | 264.29 | 156.25 | 217.50 |
|  | **Avg** | 38.23 | 27.97 | 22.06 | 166.01 | 71.50 | 25.97 | 80.39 | 93.04 | 100.30 |
|  | **Sd** | 12.21 | 11.76 | 10.99 | 9.45 | 16.46 | 5.74 | 18.33 | 17.84 | 28.88 |
| Male | **Min** | 13 | 6 | 6 | 150 | 42 | 15.4 | 39.23 | 43.63 | 42.13 |
|  | **Max** | 78 | 73 | 66 | 195 | 140 | 45.7 | 264.29 | 156.25 | 217.50 |
|  | **Avg** | 39.30 | 28.88 | 22.04 | 172.55 | 74.97 | 25.13 | 85.38 | 97.41 | 106.10 |
|  | **Sd** | 12.50 | 11.38 | 10.65 | 6.98 | 14.77 | 4.43 | 19.32 | 17.49 | 30.41 |
| Female | **Min** | 10 | 6 | 6 | 140 | 35 | 14.2 | 42.53 | 48.56 | 45.77 |
|  | **Max** | 70 | 73 | 73 | 185 | 141 | 56.5 | 140.41 | 135.04 | 202.58 |
|  | **Avg** | 37.02 | 26.95 | 22.07 | 159.53 | 68.08 | 26.78 | 74.84 | 88.18 | 94.66 |
|  | **Sd** | 11.77 | 12.09 | 11.36 | 6.72 | 17.30 | 6.69 | 15.37 | 16.96 | 26.11 |
| F31.1 | **Min** | 15 | 8 | 6 | 141 | 40 | 14.2 | 45.52 | 51.18 | 52.97 |
|  | **Max** | 75 | 67 | 56 | 195 | 141 | 56.5 | 152.42 | 139.55 | 200.00 |
|  | **Avg** | 37.88 | 26.15 | 21.42 | 164.99 | 72.61 | 26.74 | 79.23 | 92.12 | 101.80 |
|  | **Sd** | 12.22 | 10.80 | 10.22 | 9.62 | 17.97 | 6.54 | 16.93 | 17.60 | 28.15 |
| F31.2 | **Min** | 16 | 6 | 6 | 150 | 42 | 16.5 | 42.55 | 50.00 | 50.76 |
|  | **Max** | 73 | 65 | 65 | 190 | 121 | 48.5 | 140.41 | 129.29 | 184.72 |
|  | **Avg** | 38.83 | 30.72 | 24.02 | 167.13 | 71.47 | 25.55 | 80.74 | 93.78 | 100.76 |
|  | **Sd** | 11.91 | 11.04 | 11.04 | 9.50 | 15.44 | 5.11 | 16.10 | 17.07 | 26.54 |
| F31.9 | **Min** | 10 | 6 | 6 | 140 | 35 | 15.2 | 39.23 | 43.63 | 42.13 |
|  | **Max** | 67 | 73 | 73 | 188 | 140 | 51.1 | 264.29 | 156.25 | 217.50 |
|  | **Avg** | 37.46 | 28.00 | 21.53 | 165.58 | 71.18 | 25.97 | 81.20 | 93.68 | 100.88 |
|  | **Sd** | 11.62 | 12.08 | 11.13 | 9.29 | 16.51 | 5.72 | 19.85 | 17.99 | 29.97 |
| F31.3-8 | **Min** | 15 | 6 | 6 | 149 | 37 | 14.8 | 44.29 | 48.85 | 43.43 |
|  | **Max** | 78 | 67 | 66 | 185 | 93 | 33.2 | 130.65 | 134.04 | 163.27 |
|  | **Avg** | 43.41 | 24.39 | 21.43 | 169.25 | 70.14 | 24.55 | 76.84 | 88.57 | 87.63 |
|  | **Sd** | 15.97 | 12.76 | 11.53 | 8.88 | 13.41 | 4.50 | 17.42 | 19.45 | 27.60 |

Table 1S. Patient characteristics - quantitative values (795 episode)

**Table S2.** Patient characteristics - nominal and present or absent values (795 episode)

| Title | Values | Tot | M | F | F31.1 | F31.2 | F31.9 | F31.3-8 |
| --- | --- | --- | --- | --- | --- | --- | --- | --- |
| First episode | Mania | 294 | 169 | 125 | 76 | 64 | 143 | 11 |
|  | Dep | 110 | 47 | 63 | 26 | 22 | 55 | 7 |
|  | Non | 391 | 203 | 185 | 74 | 90 | 193 | 31 |
| Marriage | M | 405 | 229 | 176 | 93 | 87 | 199 | 26 |
|  | S | 258 | 139 | 119 | 59 | 57 | 126 | 16 |
|  | W | 20 | 3 | 17 | 5 | 7 | 6 | 2 |
|  | D | 90 | 38 | 52 | 16 | 23 | 47 | 4 |
|  | Non | 22 | 11 | 11 | 4 | 3 | 14 | 1 |
| Addiction | M | 83 | 61 | 22 | 18 | 24 | 32 | 9 |
|  | A | 16 | 9 | 7 | 3 | 8 | 5 | 0 |
|  | P | 42 | 30 | 12 | 8 | 14 | 18 | 2 |
|  | N | 654 | 320 | 334 | 148 | 131 | 337 | 38 |
| H_Addiction |  | 141 | 100 | 41 | 29 | 46 | 55 | 11 |
| H_Smoking |  | 288 | 202 | 86 | 67 | 66 | 133 | 22 |
| H_Alcohol consumption |  | 55 | 32 | 23 | 11 | 17 | 23 | 4 |
| H_Suicide |  | 109 | 55 | 54 | 14 | 24 | 60 | 11 |
| Resident city |  | 483 | 248 | 235 | 119 | 103 | 230 | 31 |
| Resident province |  | 692 | 365 | 327 | 158 | 152 | 340 | 42 |
| Iranian nationality |  | 784 | 416 | 368 | 175 | 176 | 385 | 48 |
| PPH_OCD |  | 40 | 24 | 16 | 6 | 9 | 22 | 3 |
| PPH_ADHD |  | 25 | 15 | 10 | 3 | 5 | 15 | 2 |
| PPH_Dep |  | 20 | 11 | 9 | 1 | 4 | 12 | 3 |
| PPH_PD |  | 10 | 9 | 1 | 0 | 1 | 8 | 1 |
| PPH_MR |  | 4 | 2 | 2 | 2 | 1 | 1 | 0 |
| PPH_PTSD |  | 7 | 6 | 1 | 2 | 0 | 4 | 1 |
| H_Trauma & head injury |  | 118 | 74 | 44 | 15 | 24 | 69 | 10 |
| H_Seizure |  | 92 | 51 | 41 | 19 | 13 | 52 | 8 |
| H_Thyroid disorder |  | 46 | 14 | 32 | 15 | 10 | 18 | 3 |
| H_Diabetes |  | 47 | 22 | 25 | 15 | 7 | 21 | 4 |
| H_Blood pressure |  | 34 | 20 | 14 | 8 | 7 | 16 | 3 |
| H_Liver disease |  | 8 | 2 | 6 | 2 | 1 | 4 | 1 |
| H_stroke |  | 3 | 2 | 1 | 1 | 0 | 0 | 2 |
| H_Kidney disease |  | 1 | 1 | 0 | 0 | 0 | 1 | 0 |
| H_Heart attack |  | 4 | 3 | 1 | 0 | 0 | 4 | 0 |
| H_Heart disease |  | 10 | 6 | 4 | 1 | 1 | 8 | 0 |
| H_CVD |  | 13 | 8 | 5 | 1 | 1 | 11 | 0 |
| H_Surgery |  | 33 | 21 | 12 | 3 | 7 | 21 | 2 |
| H_Internal disease |  | 27 | 6 | 21 | 11 | 8 | 7 | 1 |
| H_HLP |  | 15 | 9 | 6 | 7 | 3 | 4 | 1 |
| H_Neurological disease |  | 11 | 8 | 3 | 2 | 3 | 6 | 0 |
| H_Women's disease |  | 2 | 0 | 2 | 0 | 1 | 1 | 0 |
| H_Urological disease |  | 2 | 2 | 0 | 0 | 1 | 0 | 1 |
| H_Otolaryngology |  | 0 | 0 | 0 | 0 | 0 | 0 | 0 |
| H_Cancer |  | 3 | 2 | 1 | 0 | 0 | 3 | 0 |
| H_Eye disease |  | 0 | 0 | 0 | 0 | 0 | 0 | 0 |
| F.H_​​Bipolar disorder |  | 70 | 29 | 41 | 19 | 13 | 33 | 5 |
| F.H_Mental disorder 1 |  | 184 | 100 | 84 | 37 | 39 | 92 | 16 |
| F.H_Mental disorder 2 |  | 88 | 44 | 44 | 25 | 18 | 44 | 1 |
| F.H_Depression |  | 38 | 17 | 21 | 8 | 8 | 17 | 5 |
| F.H_Epilepsy |  | 10 | 3 | 7 | 2 | 3 | 4 | 1 |

**Table S3.** Patients' laboratory tests (795 episode)

| Tests | Count | Period | Min | Max | Avg | SD |
| --- | --- | --- | --- | --- | --- | --- |
| Lithium | 2150 | 12 | 0 | 3 | 0.66 | 0.29 |
| WBC (White Blood Cells) | 1563 | 20 | 2.1 | 19.7 | 7.77 | 2.59 |
| RBC (Red Blood Cells) | 1563 | 20 | 3 | 6.7 | 4.54 | 0.56 |
| Hct (Hematocrit) | 1563 | 20 | 24.4 | 53.6 | 39.79 | 4.20 |
| MCH (Mean Corpuscular Hemoglobin) | 1563 | 20 | 15.1 | 43.5 | 29.41 | 2.80 |
| MCV (Mean Corposcular Volume) | 1563 | 20 | 55.8 | 122.3 | 88.14 | 6.87 |
| MCHC (Mean Corpuscular Hemoglobin Concentration) | 1563 | 20 | 23.9 | 39.3 | 33.34 | 1.55 |
| PMN (Polymorphonuclear neutrophils) | 1563 | 20 | 33 | 89 | 64.37 | 9.89 |
| Hb (Hemoglobin) | 1562 | 20 | 5.9 | 19.6 | 13.29 | 1.69 |
| Lymph (lymphocytes) | 1562 | 20 | 6 | 63 | 27.75 | 9.20 |
| Mono (Mononucleosis) | 1560 | 20 | 1 | 18 | 4.26 | 1.73 |
| PLT (Platelet) | 1552 | 20 | 67 | 467 | 219.32 | 68.98 |
| EOS (Eosinophil) | 1550 | 20 | 1 | 11 | 3.64 | 1.56 |
| K (Potasium) | 1094 | 26 | 2.8 | 5.5 | 4.02 | 0.40 |
| Na (Sodium) | 1049 | 27 | 120 | 149 | 137.75 | 3.09 |
| Creatinine | 1006 | 10 | 0.4 | 4 | 0.98 | 0.24 |
| Urea | 1006 | 10 | 7 | 149 | 28.38 | 12.94 |
| ALT or SGPT (Alanine aminotransferase) | 1004 | 11 | 2 | 231 | 25.47 | 25.05 |
| AST or SGOT (aspartate aminotransferase) | 991 | 11 | 5 | 402 | 25.63 | 23.86 |
| ALP (Alkaline phosphatase) | 883 | 10 | 52 | 1199 | 181.88 | 79.23 |
| Ca (Calcium) | 854 | 4 | 7.7 | 10.8 | 9.34 | 0.50 |
| TSH (Thyroid stimulating hormone) | 196 | 3 | 0.1 | 50.9 | 4.41 | 7.41 |
| RDW-CV (Red blood cell distribution width) | 126 | 18 | 11.8 | 19 | 13.85 | 1.44 |
| Fe (Iron) | 53 | 2 | 18 | 156 | 78.17 | 31.97 |
| TIBC (Total iron binding capacity) | 50 | 2 | 257 | 708 | 374.30 | 78.65 |
| Folic Acid | 46 | 2 | 2.1 | 120.8 | 15.61 | 18.39 |
| MGS (Serum Magnesium) | 27 | 1 | 1.9 | 2.6 | 2.14 | 0.19 |
| Albumin | 15 | 1 | 3.5 | 5.3 | 4.28 | 0.47 |

**Table S4.** Medications taken by patients (795 episode)

| Drugs | Persons | | | | | | | | Dose | | | |
| --- | --- | --- | --- | --- | --- | --- | --- | --- | --- | --- | --- | --- |
|  | **Tot** | **M** | **F** | **F31.1** | **F31.2** | **F31.9** | **F31.3-8** | **Quantity** | | **Avg** | **SD** |  |
| Lithium300 | 795 | 420 | 375 | 177 | 177 | 392 | 49 | 32949.5 | | 41.45 | 30.77 |  |
| Valproate200.0 | 627 | 341 | 286 | 140 | 143 | 311 | 33 | 42641.2 | | 68.01 | 61.38 |  |
| Lorazepam1.0 | 524 | 264 | 260 | 116 | 120 | 261 | 27 | 14815 | | 28.27 | 25.99 |  |
| Risperidone1.0 | 519 | 288 | 231 | 108 | 120 | 265 | 26 | 34149.1 | | 65.80 | 60.73 |  |
| Haloperidol5.0 | 414 | 230 | 184 | 88 | 94 | 211 | 21 | 1943 | | 4.69 | 6.87 |  |
| Divalproex250.0 | 358 | 165 | 193 | 79 | 79 | 184 | 16 | 9664 | | 26.99 | 33.65 |  |
| Quetiapine25.0 | 339 | 189 | 150 | 66 | 70 | 177 | 26 | 34888 | | 102.91 | 118.26 |  |
| Biperiden2.0 | 336 | 198 | 138 | 67 | 72 | 182 | 15 | 6383 | | 19.00 | 20.17 |  |
| Midazolam5.0 | 328 | 181 | 147 | 77 | 74 | 159 | 18 | 1027 | | 3.13 | 4.04 |  |
| Clonazepam1.0 | 277 | 173 | 104 | 50 | 59 | 143 | 25 | 6147.8 | | 22.19 | 23.99 |  |
| Gabapentin100.0 | 235 | 163 | 72 | 40 | 57 | 122 | 16 | 12286 | | 52.28 | 72.66 |  |
| Naproxen250.0 | 231 | 142 | 89 | 46 | 60 | 108 | 17 | 6227 | | 26.96 | 32.91 |  |
| Olanzapine5.0 | 230 | 120 | 110 | 50 | 53 | 113 | 14 | 7819.1 | | 34.00 | 34.78 |  |
| Promethazine50.0 | 215 | 117 | 98 | 52 | 49 | 108 | 6 | 726.6 | | 3.38 | 3.86 |  |
| Propranolol10.0 | 162 | 85 | 77 | 39 | 33 | 74 | 16 | 5935 | | 36.64 | 35.16 |  |
| Clozapine25.0 | 131 | 94 | 37 | 25 | 31 | 66 | 9 | 6678 | | 50.98 | 68.82 |  |
| Aripiprazole5.0 | 122 | 47 | 75 | 33 | 28 | 52 | 9 | 5515 | | 45.20 | 49.30 |  |
| Folate0.4 | 94 | 30 | 64 | 22 | 27 | 41 | 4 | 5879.5 | | 62.55 | 85.68 |  |
| Clonidine0.2 | 91 | 68 | 23 | 16 | 27 | 42 | 6 | 520.1 | | 5.72 | 5.69 |  |
| Bismuth120.0 | 81 | 51 | 30 | 14 | 20 | 43 | 4 | 532 | | 6.57 | 8.45 |  |
| Metformin500.0 | 81 | 38 | 43 | 22 | 21 | 33 | 5 | 2321 | | 28.65 | 32.25 |  |
| Atorvastatin20.0 | 79 | 40 | 39 | 26 | 16 | 32 | 5 | 1371 | | 17.35 | 12.36 |  |
| Methadone5 | 68 | 54 | 14 | 10 | 17 | 35 | 6 | 4092 | | 60.18 | 74.75 |  |
| Fluphenazine25.0 | 66 | 44 | 22 | 13 | 11 | 42 | 0 | 88 | | 1.33 | 0.64 |  |
| Cyproterone50 | 58 | 25 | 33 | 14 | 13 | 29 | 2 | 889 | | 15.33 | 14.00 |  |
| Sertraline50.0 | 55 | 30 | 25 | 8 | 12 | 29 | 6 | 943 | | 17.15 | 24.32 |  |
| Carbamazepine200.0 | 51 | 21 | 30 | 12 | 12 | 22 | 5 | 1401 | | 27.47 | 33.75 |  |
| Lamotrigine25.0 | 51 | 31 | 20 | 10 | 14 | 20 | 7 | 2579 | | 50.57 | 77.33 |  |
| Omeprazole20.0 | 50 | 24 | 26 | 14 | 13 | 19 | 4 | 637 | | 12.74 | 11.04 |  |
| Flupentixol20.0 | 48 | 31 | 17 | 7 | 4 | 36 | 1 | 61 | | 1.27 | 0.44 |  |
| Haloperidol0.5 | 48 | 30 | 18 | 11 | 16 | 18 | 3 | 601 | | 12.52 | 14.88 |  |
| Pantoprazole20.0 | 48 | 29 | 19 | 9 | 13 | 23 | 3 | 753 | | 15.69 | 15.73 |  |
| Fluconazole150.0 | 45 | 2 | 43 | 12 | 12 | 21 | 0 | 134 | | 2.98 | 1.81 |  |
| Zolpidem5.0 | 45 | 40 | 5 | 6 | 7 | 32 | 0 | 268 | | 5.96 | 6.35 |  |
| Magnesium hydroxide250 | 42 | 14 | 28 | 10 | 9 | 18 | 5 | 57.3 | | 1.36 | 1.04 |  |
| Levothyroxine0.05 | 40 | 12 | 28 | 16 | 5 | 17 | 2 | 1319 | | 32.98 | 30.49 |  |
| Azithromycin250.0 | 39 | 26 | 13 | 11 | 6 | 19 | 3 | 217 | | 5.56 | 6.26 |  |
| Cefalexin500.0 | 38 | 28 | 10 | 6 | 9 | 22 | 1 | 520 | | 13.68 | 9.53 |  |
| Vitamin C1000.0 | 36 | 17 | 19 | 6 | 11 | 18 | 1 | 131.8 | | 3.66 | 5.18 |  |
| Chlorpromazine25.0 | 32 | 17 | 15 | 7 | 5 | 19 | 1 | 389 | | 12.16 | 14.91 |  |
| Citalopram20.0 | 32 | 21 | 11 | 4 | 2 | 25 | 1 | 150 | | 4.69 | 3.95 |  |
| CalciumD | 23 | 8 | 15 | 10 | 5 | 8 | 0 | 380 | | 16.52 | 15.18 |  |
| Topiramate25.0 | 22 | 10 | 12 | 7 | 6 | 8 | 1 | 1725 | | 78.41 | 88.24 |  |
| Levetiracetam500.0 | 20 | 10 | 10 | 7 | 8 | 4 | 1 | 264 | | 13.20 | 17.44 |  |
| Cobalamin1000 | 19 | 13 | 6 | 5 | 5 | 7 | 2 | 67 | | 3.53 | 3.63 |  |
| Fluoxetine10.0 | 19 | 13 | 6 | 4 | 4 | 9 | 2 | 345 | | 18.16 | 33.09 |  |
| Amitriptyline10.0 | 18 | 9 | 9 | 5 | 0 | 13 | 0 | 309.5 | | 17.19 | 22.00 |  |
| Bupropion75.0 | 18 | 9 | 9 | 3 | 0 | 9 | 6 | 201.3 | | 11.18 | 11.26 |  |
| Trihexyphenidyl2.0 | 18 | 11 | 7 | 3 | 2 | 12 | 1 | 291.5 | | 16.19 | 14.43 |  |
| Perphenazine2.0 | 14 | 5 | 9 | 6 | 3 | 4 | 1 | 176 | | 12.57 | 10.10 |  |
| Alprazolam0.5 | 13 | 9 | 4 | 3 | 0 | 8 | 2 | 56 | | 4.31 | 3.95 |  |
| Chlordiazepoxide5.0 | 13 | 11 | 2 | 3 | 1 | 9 | 0 | 81 | | 6.23 | 7.20 |  |
| Tizanidine4.0 | 13 | 11 | 2 | 2 | 3 | 5 | 3 | 97 | | 7.46 | 5.96 |  |
| Glibenclamide5.0 | 11 | 5 | 6 | 4 | 5 | 2 | 0 | 70 | | 6.36 | 6.96 |  |
| Oxybutynin5.0 | 11 | 3 | 8 | 5 | 2 | 4 | 0 | 77 | | 7.00 | 7.60 |  |
| Thiamine100.0 | 9 | 6 | 3 | 2 | 1 | 6 | 0 | 224 | | 24.89 | 29.65 |  |
| Vitamin D50000IU | 9 | 3 | 6 | 0 | 5 | 4 | 0 | 33 | | 3.67 | 2.79 |  |
| Furosemide40.0 | 7 | 3 | 4 | 3 | 0 | 4 | 0 | 84.5125 | | 12.07 | 10.90 |  |
| Amantadine100.0 | 6 | 1 | 5 | 3 | 0 | 3 | 0 | 23 | | 3.83 | 4.67 |  |
| Vitamin E100.0 | 6 | 1 | 5 | 0 | 2 | 3 | 1 | 48 | | 8.00 | 8.39 |  |
| Phenobarbital60.0 | 3 | 1 | 2 | 0 | 1 | 2 | 0 | 151.6 | | 50.53 | 44.41 |  |
